# Sevoflurane preconditioning ameliorates traumatic spinal cord injury through caveolin-3-dependent cyclooxygenase-2 inhibition

**DOI:** 10.18632/oncotarget.21142

**Published:** 2017-09-21

**Authors:** Weidong Wu, Ningxian Wei, Lihui Wang, Danhui Kong, Gang Shao, Yingchun Qin, Lixin Wang, Yansheng Du

**Affiliations:** ^1^ Danyang People's Hospital of Jiangsu Province, Danyang, Jiangsu, PR China; ^2^ School of Medicine, Southeast University, Nanjing, Jiangsu, PR China; ^3^ School of Medicine, Indiana University, Indianapolis, Indiana, United States

**Keywords:** sevoflurane preconditioning, traumatic spinal cord injury, COX-2, Cav-3, anaesthesia

## Abstract

Acute traumatic spinal cord injury (tSCI) results in a lifetime of paralysis associated with a host of medical complications. The developing secondary complications of tSCI may result in further chronic neurodegenerative diseases. Sevoflurane preconditioning (SF-PreCon) has shown guaranteed protective effects in myocardial or cerebral ischemic/reperfusion injury. However, the role of SF-PreCon in tSCI still remains to be elucidated. Here, we found that SF-PreCon ameliorated the developing secondary complications through reducing the apoptosis rate and the secretion of inflammatory cytokines in injured spinal cord tissues, and therefore enhancing the recovery after tSCI. Notably, we demonstrated that SF-PreCon ameliorates tSCI through inhibiting Cycloxygenase-2 (COX-2). Importantly, we verified that SF-PreCon inhibits the expression of COX-2 and reduces the apoptosis rate after tSCI via the induction of Caveolin-3 (Cav-3). Taken together, our results suggest that SF-PreCon ameliorates tSCI via Cav-3-dependent COX-2 inhibition and provide an economical and practical method against the secondary injury after tSCI.

## INTRODUCTION

Traumatic spinal cord injury (tSCI) occurs from external impacts such as motor vehicle collisions, sports accidents, falls, or violence and has two phases: a primary injury phases and a secondary injury phases. Acute tSCI firstly results in a lifetime of paralysis associated with a host of medical complications. After the primary spinal cord injury, the injured area was infiltrated with neutrophils, and the secretion of cytokines attracts other inflammatory cells, that would aggravate the local injury, termed secondary injury [1]. Secondary injury is the main obstacle for the recovery of tSCI, which is induced by the change of the niche microenvironment, that leading to a further tissue ischemia, hypoxia, inflammation aggravate, and then forming a vicious circle. The secondary injury may result in further chronic neurodegenerative diseases [2]. However, the efficient therapies against the secondary injury of tSCI remain urgently to be explored.

Based on the reality of limited self-repairing ability in the injured brain or spinal cord, recently developed therapies have focused on either protecting injured neurons or decreasing neuronal apoptosis within the injured region. Interestingly, previous studies have reported that the preconditioning of sevoflurane, one of the most commonly used volatile anesthetic agents with the fastest onset and offset in modern anesthesiology, exerts direct neuroprotective effects in cerebral ischemia [3]. Although sevoflurane preconditioning (SF-PreCon) has been reported to protect against ischemia/reperfusion injury of multiple organs, including lung and heart [4, 5], whether SF-PreCon shows protective effects in tSCI is still unknown.

Cyclooxygenase-2 (COX-2) is a conditional enzyme, which is only induced and expressed in the case of inflammatory damage [6]. Cytokines that are secreted by inflammatory cells, including IL-1, TNF-α and interferon, can induce the expression of COX-2 [7]. Inhibition of COX-2 after transient cerebral ischemia can reduce blood-brain-barrier damage, vascular edema and leukocyte infiltration [8]. Previous studies have reported that the protective effects of SF-PreCon on myocardial ischemia injury or acute lung injury were achieved through the inhibition of COX-2 [9, 10]. Recently, SF-PreCon was reported to attenuate myocardial ischemia/reperfusion injury via cavelin-3 (Cav-3)-dependent COX-2 inhibition [5]. All of these studies strongly indicate the involvement of Cav-3/COX-2 axis in the effects of SF-PreCon on tSCI.

Here, we found that SF-PreCon could attenuate apoptosis, reduce the expression of inflammatory cytokines and then improve neurological assessment scores after tSCI. Moreover, we demonstrated that SF-PreCon attenuates tSCI through inhibiting COX-2, which is a Cav-3-dependent manner.

## RESULTS

### SF-PreCon improved neurological assessment scores after tSCI

**Figure 1:**
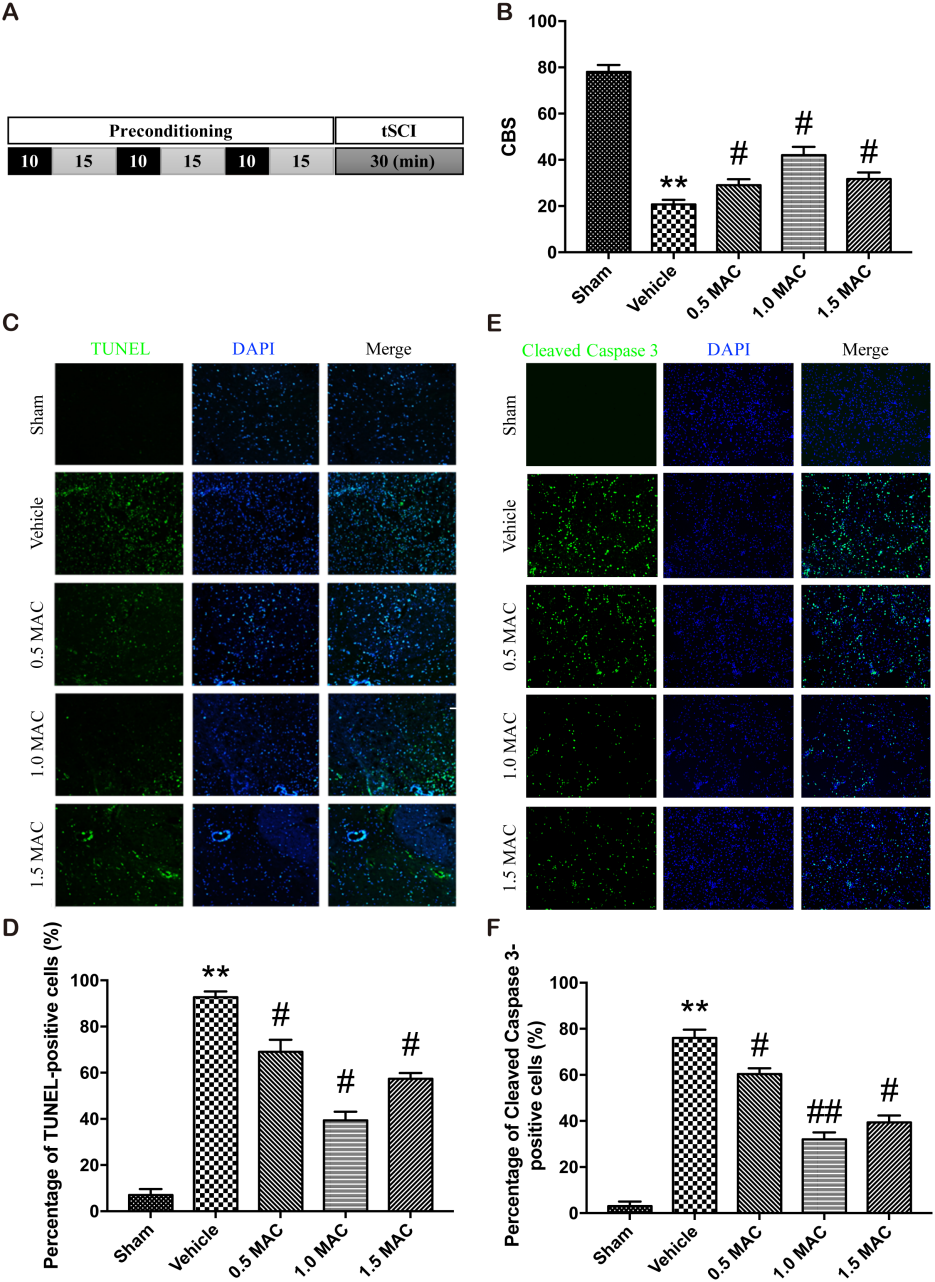
SF-PreCon efficiently improves the neurological assessment scores and attenuates the apoptosis after tSCI **(A)** Schematic illustration of SF-PreCon protocol used in this experiment. **(B)** Comparison of the mean CBS score in the five groups. Data are mean ± SEM; ^**^ *P* < 0.01 versus the Sham group; ^#^ *P* < 0.05 versus the Vehicle group; unpaired two-tailed Student's *t*-test. *n* = 5 per group. **(C)** Representative slides of TUNEL staining of the spinal cord sections. Magnification = ×100. **(D)** Percentage of TUNEL-positive cell in C were calculated. Data are mean ± SEM; ^**^ *P* < 0.01 versus the Sham group; ^#^ *P* < 0.05 versus the Vehicle group; unpaired two-tailed Student's *t*-test. *n* = 5 per group. **(E)** Representative slides of Cleaved Caspase 3 staining of the spinal cord sections. Magnification = ×100. **(F)** Percentage of Cleaved Caspase 3-positive cell in E were calculated. Data are mean ± SEM; ^**^ *P* < 0.01 versus the Sham group; ^#^ *P* < 0.05 or ^##^ *P* < 0.01 versus the Vehicle group; unpaired two-tailed Student's *t*-test. *n* = 5 per group.

Previous studies have showed that SF-PreCon protects against ischemia/reperfusion injury of multiple organs, including lung, heart and brain. To evaluate the effects of SF-PreCon on tSCI, adult female rats were assigned to different groups as described below. The sham group: five adult female SD rats were subjected to sham operations without tSCI. The vehicle group: five adult female SD rats were subjected to tSCI without SF-PreCon. The SF-PreCon group with tSCI: five adult female SD rats were subjected to tSCI after SF-PreCon, within which rats were exposed to 3 cycles of 10-minute exposure to 0.5 minimum alveolar concentration (MAC) sevoflurane interspersed with 15 minutes of washout (Figure 1A). Then, the time course of neurological scores was assessed by means of the combined behavioral score (CBS) score after tSCI. Results showed that there are significant decreases in average CBS scores (relative to baseline) in vehicle group (Figure 1B), confirming the usefulness of this assessment system. And the average CBS scores after tSCI were improved in rats preconditioned with sevoflurane (Figure 1B), suggesting SF-PreCon enhanced the recovery after tSCI.

To determine the most suitable concentration of sevoflurane that used for preconditioning, we employed another two SF-PreCon groups with 1.0 MAC or 1.5 MAC sevoflurane treatment, and found that 1.0 MAC SF-PreCon showed the highest CBS score (Figure 1B).

### Apoptosis of cells following tSCI is reduced by SF-PreCon

An efficient and applicable therapy against the secondary injury after tSCI should reduce the apoptosis of neurons in the injury spinal cord. Thus we examined the occurrence of apoptosis in the injured spinal cord after tSCI using TUNEL assay. Spinal cord sections from injured and treated groups were analyzed for the number of TUNEL-positive cells. Results showed that the percentage of TUNEL-positive cells was obviously increased after tSCI, and this increase was significantly compromised by SF-PreCon (Figure 1C). Moreover, 1.0 MAC SF-PreCon group showed the lowest percentage of TUNEL-positive cells among the three SF-PreCon groups (Figure 1D), indicating the most efficient dosage for the attenuation of tSCI. Notably, the immunofluorescence of Cleaved Caspase 3, a critical executioner of apoptosis, further confirmed that SF-PreCon reduced the occurrence of apoptosis following tSCI (Figure 1E and 1F).

### SF-PreCon resulted in reduced expression of inflammatory cytokines after tSCI

**Figure 2:**
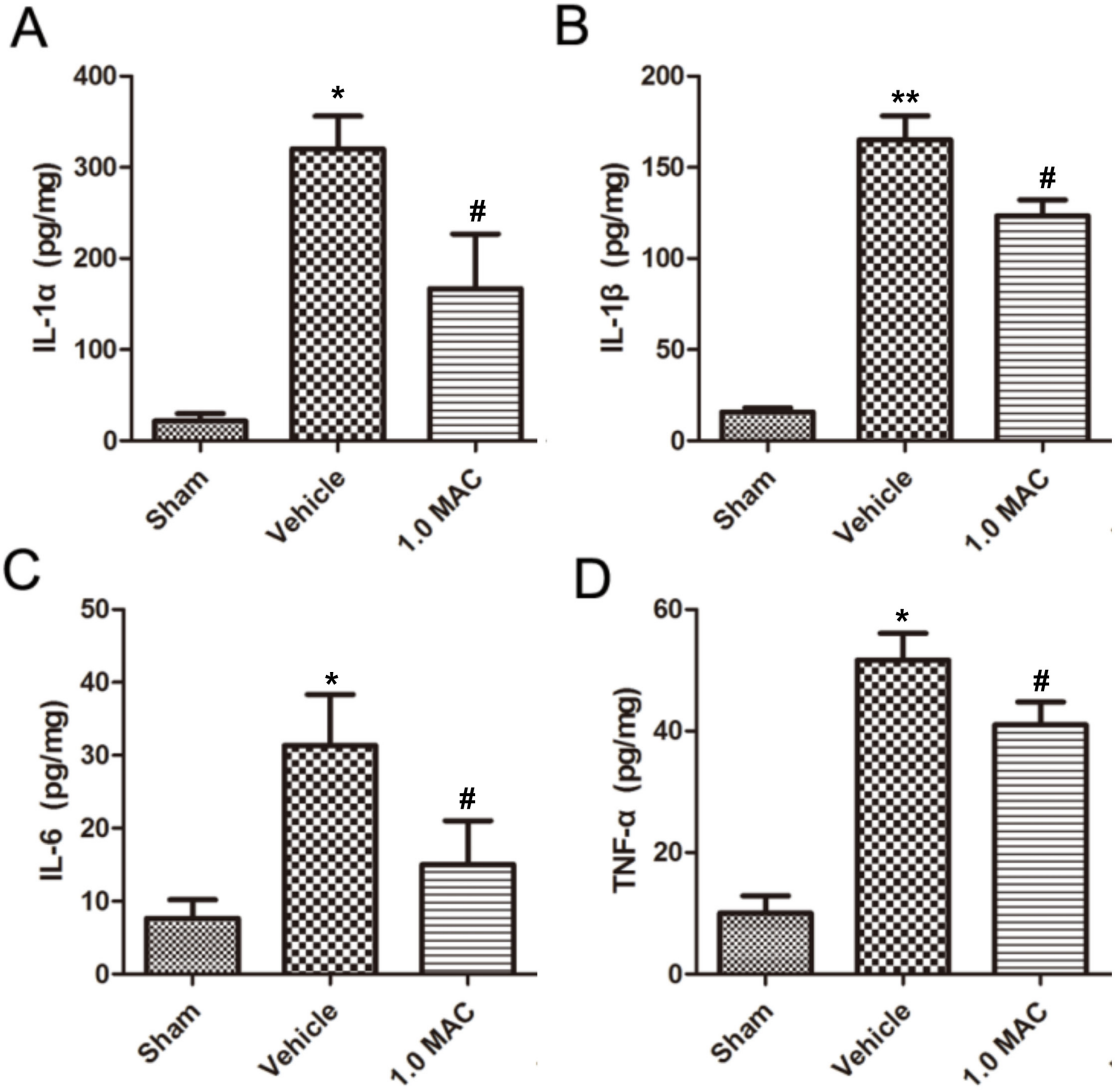
SF-PreCon significantly inhibits the secretion of inflammatory cytokines in the injured spinal cord tissues after tSCI ELISA of cytokines IL-1α **(A)**, IL-1β **(B)**, IL-6 **(C)**, and TNF-α **(D)** in tSCI rats with or without 1.0 MAC SF-PreCon. Data are mean ± SEM; ^*^ *P* < 0.05 versus the Sham group; ^#^ *P* < 0.05 versus the Vehicle group; unpaired two-tailed Student's *t*-test. *n* = 5 per group.

Traumatic lesions dramatically reduce life quality and lead to severe and often fatal impairments, largely because the low capacity for regeneration. And the acute inflammatory response that takes place rapidly after tSCI is put forward as one of the major elements affecting the regenerative outcome [11]. To examine whether SF-PreCon modulates the inflammatory response, cytokine levels were examined in spinal cord tissues using ELISA assay. The results showed that IL-1α, IL-1β, IL-6, and TNF-α were dramatically increased in tSCI rats of vehicle group compared with the rats of sham group, whereas all of these increases were significantly compromised by 1.0 MAC SF-PreCon (Figure 2A-2D). Together, these results demonstrated that SF-PreCon efficiently ameliorates the secondary injury after tSCI.

### SF-PreCon attenuates tSCI through Cav-3 dependent COX-2 inhibition

**Figure 3:**
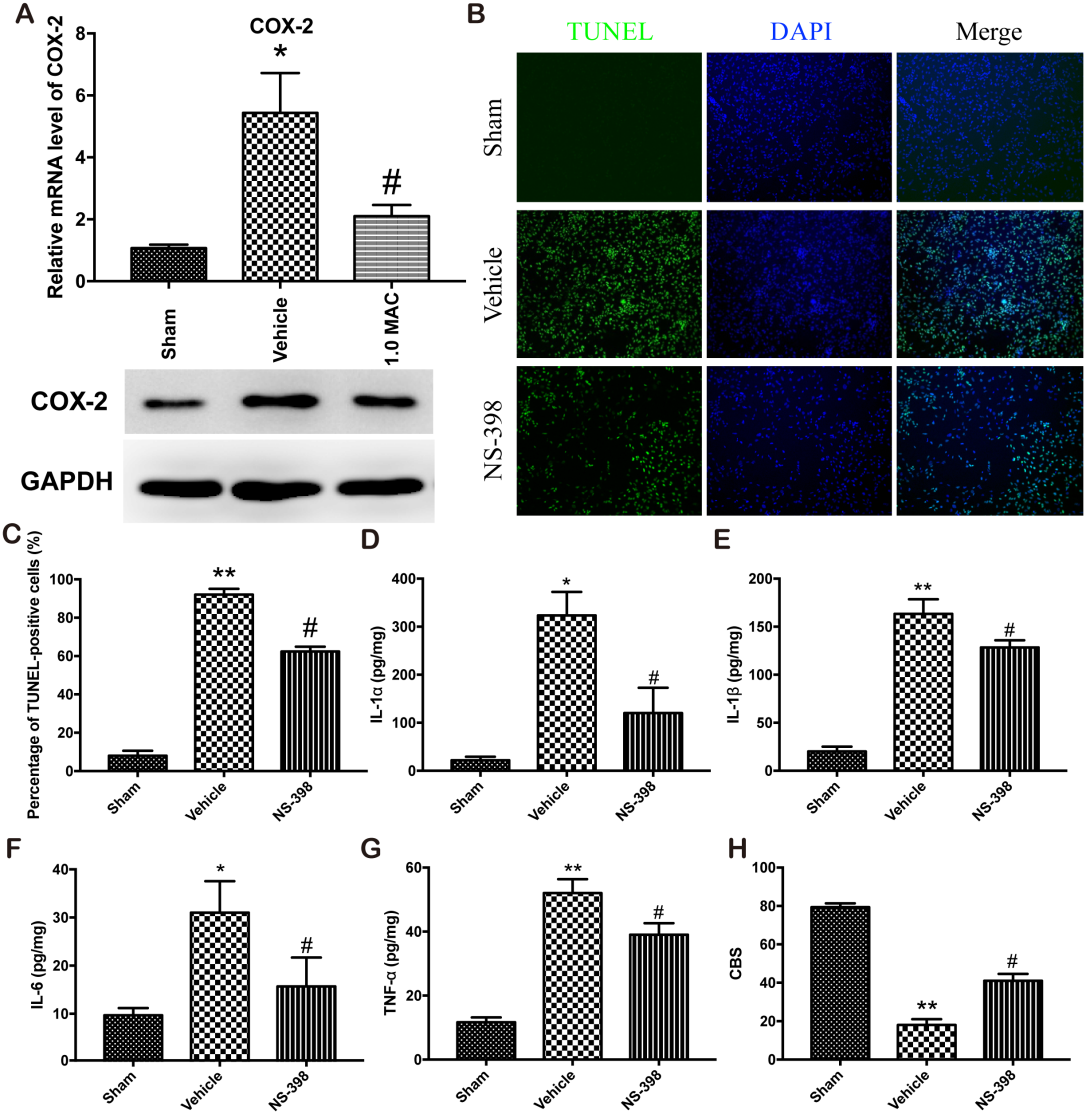
SF-PreCon attenuates the secondary injury after tSCI through the inhibition of COX-2 expression **(A)** Real-time PCR and western blotting assay for COX-2 mRNA and protein levels in tSCI rats with or without 1.0 MAC SF-PreCon. Data are mean ± SEM; ^*^ *P* < 0.05 versus the Sham group; ^#^ *P* < 0.05 versus the Vehicle group; unpaired two-tailed Student's *t*-test. *n* = 5 per group. **(B)** Representative slides of TUNEL staining of the spinal cord sections with or without NS-398 treatment. Magnification = ×100. **(C)** Percentage of TUNEL-positive cell in B were calculated. Data are mean ± SEM; ^**^ *P* < 0.01 versus the Sham group; ^#^ *P* < 0.05 versus the Vehicle group; unpaired two-tailed Student's *t*-test. *n* = 5 per group. **(D-G)** ELISA of cytokines IL-1α (D), IL-1β (E), IL-6 (F), and TNF-α (G) in tSCI rats with or without NS-398 treatment. Data are mean ± SEM; ^*^ *P* < 0.05 or ^**^ *P* < 0.01 versus the Sham group; ^#^ *P* < 0.05 versus the Vehicle group; unpaired two-tailed Student's *t*-test. *n* = 5 per group. **(H)** The mean CBS score in tSCI rats was improved by NS-398 treatment. Data are mean ± SEM; ^**^ *P* < 0.05 versus the Sham group; ^#^ *P* < 0.05 versus the Vehicle group; unpaired two-tailed Student's *t*-test. *n* = 5 per group.

Numerous studies have showed that SF-PreCon attenuates myocardial ischemia injury or acute lung injury through the inhibition of COX-2 [9, 10]. To examine whether COX-2 is responsible for the protective effect of SF-PreCon on tSCI, we first detect the expression level of COX-2 in the spinal cord tissues. The mRNA and protein level analysis showed that tSCI dramatically elevated the expression level of COX-2, while SF-PreCon clearly hindered the elevation (Figure 3A). To further confirm the direct involvement of COX-2, we subjected adult female SD rats to spinal cord injury with NS-398 to inhibit the activity of COX-2, and found that the inhibition of COX-2 greatly reduced the apoptosis rate and the excessive inflammatory response, and then potentiated the neurological assessment scores after tSCI (Figure 3B-3H). Collectively, these results suggested that SF-PreCon ameliorates tSCI through the inhibition of COX-2.

**Figure 4:**
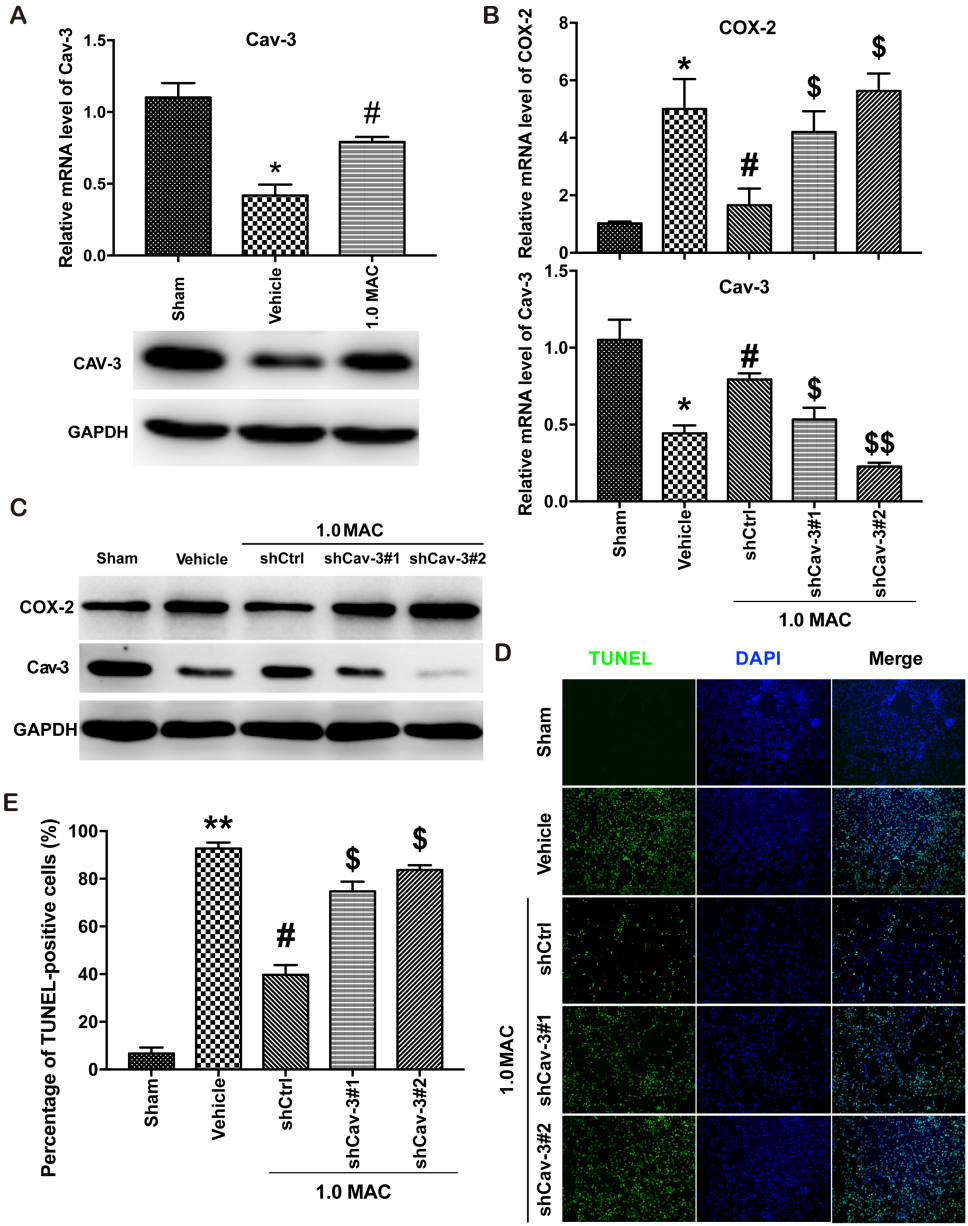
SF-PreCon attenuates tSCI through Cav-3 dependent COX-2 inhibition **(A)** Real-time PCR and western blotting assay for Cav-3 mRNA and protein levels in tSCI rats with or without 1.0 MAC SF-PreCon. Data are mean ± SEM; ^*^ *P* < 0.05 versus the Sham group; ^#^ *P* < 0.05 versus the Vehicle group; unpaired two-tailed Student's *t*-test. *n* = 5 per group. **(B)** Real-time PCR assay for COX-2 and Cav-3 mRNA levels after Cav-3 knockdown with 1.0 MAC SF-PreCon. Data are mean ± SEM; ^*^ *P* < 0.05 versus the Sham group; ^#^ *P* < 0.05 versus the Vehicle group; $ *P* < 0.05 or $$ *P* < 0.01 versus the shCtrl group; unpaired two-tailed Student's *t*-test. *n* = 5 per group. **(C)** Western Blotting assay for COX-2 and Cav-3 protein levels after Cav-3 knockdown with 1.0 MAC SF-PreCon. **(D)** Representative slides of TUNEL staining of the spinal cord sections after Cav-3 knockdown with 1.0 MAC SF-PreCon. Magnification = ×100. **(E)** Percentage of TUNEL-positive cell in D were calculated. Data are mean ± SEM; ^**^ *P* < 0.01 versus the Sham group; ^#^ *P* < 0.05 versus the Vehicle group; $ *P* < 0.05 versus the shCtrl group; unpaired two-tailed Student's *t*-test. *n* = 5 per group.

Recently, SF-PreCon was reported to inhibit COX-2 expression through the induction of Cav-3 in myocardial ischemia/reperfusion injury [5]. To verify whether the inhibition of COX-2 is Cav-3 dependent, we first examined the expression level of Cav-3, and found that tSCI significantly inhibited the expression of Cav-3 whereas SF-PreCon efficiently improved the Cav-3 expression (Figure 4A). Next, we used two efficient shRNAs against the coding region of Cav-3 to knockdown Cav-3 in the injured spinal cord tissues via adenovirus delivery system. And the results showed that Cav-3 knockdown clearly disrupted the SF-PreCon-inhibited expression of COX-2, and impaired the protective effects of SF-PreCon on tSCI (Figure 4B-4E). Taken together, our data supported that SF-PreCon ameliorates tSCI through Cav-3 dependent COX-2 inhibition.

## DISCUSSION

In this study, we found that SF-PreCon attenuated the apoptosis, reduced the expression of inflammatory cytokines in the injured spinal cord tissues, and therefore enhanced the recovery after tSCI. Importantly, we demonstrated that SF-PreCon ameliorates tSCI through inhibiting COX-2 in a Cav-3-dependent manner. Collectively, our data shed light on the protective role of SF-PreCon in attenuating the developing secondary complications after tSCI via Cav-3-dependent COX-2 inhibition.

tSCI is a devastating neurological disorder that results in irreversible neurological impairment of the motor, sensory and/or autonomic nervous systems. And the developing secondary complications will aggravate the injury of tSCI. There are emerging evidences that the systemic processes of care can improve patient outcomes and decrease the cost of care. And minimizing the risk of developing secondary injury of tSCI will be the most underlying way to improve the patient's health status and decrease both the demand and cost of medical intervention. Here we reported an economical and practical therapy that could efficiently attenuate the apoptosis of the injured spinal cord nerve cells, and protect these cells from the vandalization by inflammatory cytokines via inhibited the secretion of the inflammatory cytokines. Eventually, the neurological assessment scores were significantly improved.

Sevoflurane, a volatile anesthetic with the unique clinical characteristics of rapid recovery time with little emergence agitation, is suitable for both induction and maintenance of anesthesia and as a result is the most frequently used anesthetic, clinically. The neuroprotective effects of SF-PreCon have been well documented in several ischemia/reperfusion models, including myocardial ischemia/reperfusion injury, hepatic ischemia/reperfusion injury, cerebral ischemia/reperfusion injury, and the ischemia/reperfusion injury in lung transplantation [3–5, 12]. Moreover, SF-PreCon has also been reported to ameliorate neuronal deficits and induce rapid ischemic tolerance after spinal cord ischemia/reperfusion injury [13]. However, whether SF-PreCon shows neuroprotective effects in tSCI still remains to be explored. In this study, we strongly demonstrated that the preconditioning of sevoflurane efficiently ameliorates the developing secondary injury after tSCI, which will facilitate the improvement of patient's health status.

Endogenous cells (neurons and microglia) in the human spinal cord, not the blood-borne leukocytes, contribute to the early production of IL-1α, IL-1β, IL-6, and TNF-α in the post-traumatic inflammatory response, and microglia are involved the early response to traumatic axonal injury. TNF-α is rapidly expressed in the injured area, and promotes the migration of granule cells to the spinal cord and the production of inflammatory cytokines [14, 15]. It has been reported that the inhibition of COX-2, which catalyzes the oxidation of arachidonic acid and finally generates a series of prostaglandin, can alleviate the inflammation responses and reduce the damage of neurons in the injured nerve in various ischemia/reperfusion injury models [8, 16]. In this study, we observed the obviously decreased expression level of COX-2 and reduced secretion of the inflammatory cytokines in the SF-PreCon group after tSCI. Moreover, selective inhibition of COX-2 with NS-398 diminished the inflammation response and improved the neurological assessment scores following tSCI. Therefore, our study of the protective mechanism of sevoflurane preconditioning in tSCI provides experimental and theoretical basis for the clinical anesthesia of tSCI.

Cav-3 was expressed constitutively in some astrocytes, but not in endothelial cells; its immunoreactivity was increased in reactive astrocytes in EAE lesions [17]. Many signaling molecules compartmentalize within cardiomyocyte caveolae and interact with the scaffolding domain of Cav-3 [18]. Recently, it has been demonstrated that the cardioprotective effects of isoflurane bolus administration against myocardial ischemia are abolished when caveolae formation is disrupted or Cav-3 is knocked out [19]. Our study provides evidence that SF-PreCon induces the expression of Cav-3 to decrease the expression of COX-2 after tSCI. And the neuroprotective effect of SF-PreCon was impaired when Cav-3 is knocked down, indicating a Cav-3-dependent manner. Taken together, our data demonstrated that SF-PreCon reduces the apoptosis rate and the secretion of inflammatory cytokines in injured spinal cord tissues, and therefore ameliorates the developing secondary complications after tSCI through Cav-3 dependent COX-2 inhibition. Finally, SF-PreCon might be an economical and practical method for facilitating the recovery from tSCI.

## MATERIALS AND METHODS

### Traumatic spinal cord injury of rat model

All animal procedures were approved by the Institutional Animal Care and Use Committee at the Danyang People's Hospital of Jiangsu Province. Moderate tSCI was induced using the weight drop device as reported previously [20–22]. Adult female Sprague-Dawley rats (200–250 g) were obtained from Slaccas Laboratory Animal, (Shanghai, China). To create a contusive tSCI, the rats received ketamine (0.25 mL/kg, 100 mg/mL). The laminectomy was performed to expose the spinal cord at T8, and a moderate tSCI was created using a weight-drop device, with a 10 g weight dropped 2.5 cm. Rats were acclimatized for at least 7 days prior to the operation and were bred in standard cages on a 12 h light/dark cycle with free access to food and water. The preconditioning of sevoflurane was applied according to the previous study [5]. NS-398 (Selleck, S8433, USA) was dissolved in 0.5 mL of 1:1 (v/v) DMSO/saline such that the final dose delivered was 5 mg/kg NS-398 intraperitoneally [16]. For interfering with the expression of Cav-3 in injured spinal cord tissues via adenovirus delivery system, two shRNAs targeting the CDS of Cav-3 mRNA were designed (shCav-3#1: GACCGAAGAGCACACAGATCT; shCav-3#2: GGGTGAGCTACACCACTTTCA) and cloned to pAd-hU6-CMV-puromycin cloning vector. A scramble shRNA (shCtrl: CCTAAGGTTAAGTCGCCCTCG) was also designed as a control.

### Neurological scoring

The neurological function of each group was evaluated according to a modified Tarlov method and combined behavioral scoring (CBS) 1 week after surgery (tSCI or/and SF-PreCon). [22–24]

### TUNEL assay and immunofluorescence assay

A TUNEL (terminal deoxynucleotidyl-transferasemediated dUTP nick-end labeling) assay is the most commonly used technique for examining apoptosis via DNA fragmentation. *In situ* detection of apoptosis in spinal cords was performed through staining using a TUNEL kit from Roche (Mannheim, Germany) to modify genomic DNA utilizing terminal deoxynucleotidyl transferase (TdT), according to the manufacturer's instructions.

For immunofluorescence studies, the sections were washed three times in PBS and incubated in anti-Cleaved Caspase 3 rabbit polyclonal antibody (Cell Signaling Technology, USA) for 3 h at room temperature, and then incubated in FITC-conjugated anti-rabbit secondary antibody (Invitrogen, USA) for 1 h at room temperature. Then, cell nuclei were labeled with DAPI (Santa Cruz Biotechnology, USA), and examined under a laser scanning spectral confocal microscope by two independent observers.

Average percentage of TUNEL-positive or Cleaved Caspase 3-positive motor neurons in the anterior spinal cord of the three sections were counted for comparisons among the groups. Positively stained fluorescein-labeled cells were visualized and photographed by fluorescence microscopy.

### ELISA analysis

Spinal cords were collected, homogenized, and centrifuged to obtain tissue for ELISA. Inflammatory cytokine (IL-1α, IL-1β, IL-6, TNF-α) content were determined using ELISA kits (Dakewei Bio, China). According to the manufacturers’ instructions, absorbance (A) was quantified at λ = 450 nm. The IL-1β content of each sample was calculated based on a standard curve. IL-1α, IL-1β, IL-6, and TNF-α concentrations were expressed in pg/mg protein.

### Western blotting

Spinal cords were harvested, homogenized, and lysed with protease inhibitor-containing RIPA buffer. The concentrations of protein lysate were calculated using the Pierce BCA Protein Assay Kit (Thermo Scientific, USA). A total of 20 μg ~ 30 μg of proteins was separated by SDS-PAGE, transferred to nitrocellulose membranes, and blotted with the following primary antibodies: anti-COX-2 (ab52237, abcam, USA), anti-Cav-3 (ab2912, abcam, USA) and anti-GAPDH (G9545, Sigma, USA). After overnight incubation, the membranes were blotted with HRP- conjugated secondary antibody (Jackson ImmunoResearch, USA), and visualized using Clarity ECL Westerm Blotting Substrate (Bio-rad, USA).

### RNA isolation and real-time PCR

RNAs were extracted from cells using TRIzol (Invitrogen, USA) kit according to the manufacturer's instructions. Subsequently, total RNA was reverse transcribed using SuperScript III reverse transcriptase (Invitrogen, USA). Real-time PCRs were then performed in ABI PRISM7500 system (Applied Biosystems, USA), according to the manufacturer's instructions. The expression level of each gene was normalized by GAPDH and reported as relative levels. The primers for mRNAs real-time PCR were shown as below. COX-2: forward primer 5′-TGTATGCTACCATCTGGCTTCGG- 3′ and reverse primer 5′- GTTTGGAACAGTCGCTCGTCATC-3′; Cav-3: forward primer 5′- GGCACGGATCATCAAGGACA- 3′ and reverse primer 5′- GTGTAGCTCACCCTCCACAC-3′; GAPDH: forward primer 5′- GAAGGTCGGTGTGAACGGAT- 3′ and reverse primer 5′- ACCAGCTTCCCATTCTCAGC-3′.

### Statistical analysis

All data were expressed as mean ± SEM of at least three independent experiments. All variables measured in this study were normally distributed. Statistical significance was determined using unpaired two-tailed Student's *t*-test. A P value of <0.05 was considered statistically significant.

## Abbreviations

tSCI: traumatic spinal cord injury
SF-PreCon: sevoflurane preconditioning
COX-2: cycloxygenase-2
Cav-3: Caveolin-3
MAC: minimum alveolar concentration
CBS: combined behavioral score
